# GRANDPARENTS’ CAREGIVING STRESS AND RESILIENCE AMID THE COVID-19 PANDEMIC IN HONG KONG

**DOI:** 10.1093/geroni/igae098.1770

**Published:** 2024-12-31

**Authors:** Vera Mun Yu Tang, Sonia Kin Lai Chan

**Affiliations:** The University of Hong Kong, Hong Kong, China (People’s Republic); The University of Hong Kong, Hong Kong, China (People’s Republic)

## Abstract

Informal care provided by grandparents became more crucial to children with parents unable to look after them at home during COVID-19 lockdown when formal childcare services were suspended and schools were closed. However, there is limited understanding of grandparents’ experiences of caregiving to grandchildren beyond the early COVID-19 waves (i.e., from July 2020 onward). This study applies Lazarus and Folkman’s transactional theory of stress and coping to understand grandparents’ primary appraisal of stressor, coping strategies, and perceived self-efficacy (i.e., secondary appraisal) in providing childcare during the third and forth COVID-19 waves in Hong Kong. Individual interviews were conducted with 21 grandparents aged 45 or above, who had been taking care of grandchildren aged 0 to 15 during the COVID-19 pandemic or six months before the outbreak in Hong Kong. A coding team performed content analysis to identify emergent themes across cases. This study reveals that grandparents’ stress appraisal of pandemic in childcare as threat to harm their grandchildren’s health and development, and as challenge to effective communication with grandchildren and adult children. They initiated problem-focused coping strategies to ameliorate the impact, including lifestyle changes through practicing hygiene and sanitation to protect their grandchildren from infection, performing multiple roles as teacher, playmate, carer and activity facilitator, and learning new communication technologies. They felt pride in their efforts and abilities to ensure grandchildren’s safety and whole-person development, and maintain family stability amid an unprecedented health crisis. These findings suggest that positive caregiving appraisal is crucial for grandparental resilience in stressful times.

